# Which Sternal Closure Technique is More Beneficial in Cardiac Surgery: Simple Wire, Figure-of-8, or Their Combination?

**DOI:** 10.21470/1678-9741-2025-0011

**Published:** 2025-10-15

**Authors:** Osman Fehmi Beyazal, Mehmed Yanartaş

**Affiliations:** 1 Department of Cardiovascular Surgery, Istanbul Basaksehir Cam and Sakura City Hospital, Istanbul, Turkiye

**Keywords:** Sternum, Surgical Wound Dehiscence, Wound Infection, Cardiac Surgery, Decompression.

## Abstract

**Introduction:**

The aims of this study are to compare sternal closure techniques (single, figure-of-8, and combined use) in patients undergoing cardiac surgery and to investigate their relationship with postoperative sternal complications.

**Method:**

Between 2023 and 2024, 645 patients (470 males; mean age 58.5 ± 11.1 years) who underwent cardiac surgery were evaluated. The patients were divided into three groups: Group 1, simple wire (n = 141); Group 2, figure-of-8 (n = 224); and Group 3, combination of these two techniques (n = 280). Preoperative and perioperative data, postoperative complications, and sternal complications were compared between these groups.

Result: The distribution ratio of the groups is 141 (22%), 224 (35%), and 280 (43%) in Groups 1, 2, and 3, respectively. There was no significant difference between the groups regarding basic demographic characteristics, comorbidities, and operative data. There was no difference between the groups in terms of postoperative exploration, delayed chest closure, subxiphoid decompression, superficial sternal wound infection (SSWI), deep sternal wound infection (DSWI), vacuum-assisted closure usage, intubation time, intensive care unit stay, and mortality. The hospital stay was found to be shorter in Group 3 compared to the other groups (median 8 days - 7 days, P = 0.02).

**Conclusion:**

In patients undergoing cardiac surgery, we found no difference in sternal complications (DSWI, SSWI) between the three most commonly used closure techniques (simple wire, figure-of-8, and their combination). We found that the length of hospital stay was shorter in patients with the combined technique than in the other two techniques.

## INTRODUCTION

**Table:** 

Abbreviations, Acronyms & Symbols				
ALT	= Alanine aminotransferase		ICU	= Intensive care unit
AST	= Aspartate aminotransferase		IQR	= Interquartile range
AVR	= Aortic valve replacement		LITA	= Left internal thoracic artery
BITA	= Bilateral internal thoracic artery		LVAD	= Left ventricular assist device
BMI	= Body mass index		MANOVA	= Multivariate Analysis of Variance
BSA	= Body surface area		MRA	= Mitral ring annuloplasty
CABG	= Coronary artery bypass grafting		MVR	= Mitral valve replacement
COPD	= Chronic obstructive pulmonary disease		PVR	= Pulmonary valve replacement
CPB	= Cardiopulmonary bypass		RBC	= Red blood cells
CRP	= C-reactive protein		TRA	= Tricuspid ring annuloplasty
CVA	= Cerebrovascular accident		TVR	= Tricuspid valve replacement
DM	= Diabetes mellitus		SSWI	= Superficial sternal wound infection
DSWI	= Deep sternal wound infection		VAC	= Vacuum-assisted closure
EuroSCORE	= European System for Cardiac Operative Risk Evaluation		WBC	= White blood cells
FFP	= Fresh frozen plasma		XCL	= Cross-clamping

Although minimally invasive methods are developing in cardiac surgery today, median sternotomy is still considered the gold standard method for many cardiac operations. Sternal complications are one of the most important causes of morbidity and mortality after cardiac surgery. Sternal wound complications are seen in 0.5 - 6.1% of patients after cardiac surgery, and deep sternal wound infections (DSWI) can cause mortality between 14 and 47%^[1]^. The most common associated factors are chronic obstructive pulmonary disease (COPD), obesity, diabetes mellitus (DM), renal failure, immunosuppression, osteoporosis, and previous sternotomy^[2]^. Sternotomy closure techniques may also be associated with sternal complications.

For many patients who do not have a risk factor for sternal complications, steel wires are the most commonly used products. Different closure methods such as single, double, figure-of-8, or their combination can be preferred for sternum closure with steel wires. In these techniques, the location of the wires relative to the sternum, the contact surface area, and the forces acting on the sternum differ. Accordingly, postoperative sternal complication rates may vary. Although studies are comparing these techniques in the literature, no definitive consensus has been reached on which technique to use. In the meta-analysis by Shafi et al.^[3]^, it was shown that the double wire or figure-of-8 technique was more beneficial than the single wire in terms of sternal instability. In the study by Almdahl et al.^[4]^, sternal closure with figure-of-8 was shown to be better than closure with simple interrupted wires. In the study of Tekümit et al.^[5]^, there was no difference between figure-of-8 and simple wire in terms of sternal dehiscence.

In our clinic, steel wire is used as a standard for patients who do not have significant risk factors for sternal complications. As a closure technique, single, figure-of-8, or a combination of these techniques are used. Although studies are comparing single and figure-of-8 closure methods in the literature, there is not enough data on the combined use of these techniques or the comparison of these three techniques. The aims of this study are to compare sternal closure techniques (single, figure-of-8, and combined use) in patients undergoing cardiac surgery and to investigate their relationship with postoperative sternal complications.

## METHODS

This study was designed as a retrospective single-center observational study with a total of 800 patients. All patients over the age of 18 years who underwent cardiac surgery in the Cardiovascular Surgery Clinic of Istanbul Basaksehir Cam and Sakura City Hospital between January 2023 and April 2024 were included in the study. Emergency surgeries, those who underwent surgery due to aortic dissection, those who underwent surgery with minimally invasive techniques, those who underwent mini-sternotomy, those who used sternal Cable System (Pioneer Surgical Technology Inc., Marquette, Michigan, United States of America) or plate, and those who received extracorporeal membrane oxygenation or intra-aortic balloon pump in the perioperative period were excluded from the study. Following the inclusion and exclusion criteria, 645 patients were included in the study. The patients were divided into three groups: Group 1, simple wire group (n = 141); Group 2, figure-of-8 group (n = 224); and Group 3, combination of the first two techniques (n = 280).

**Fig. 1:**
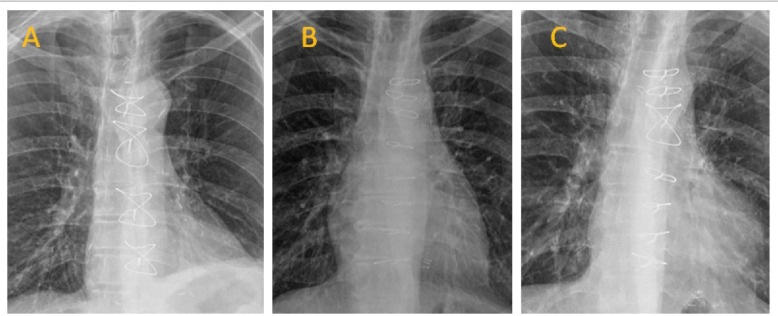
Chest radiographs of sternal closure techniques. A) Figure-of-8; B) simple wire; C) combined technique.

For the sternum closure technique, one of the three methods described was used according to the surgeon's preference. In patients who underwent simple wiring, seven or eight single wires were frequently used, depending on the length of the sternum. In patients who underwent figure-of-8, four wires were frequently used. In patients in Group 3, the first two wires were single, then one figure-of-8 at the level of the manubrium sterni, and then four single wires were used (Figure 1). Number 6 steel wires (Covidien Inc., Mansfield, United States of America) were used in all groups. Bone wax was applied routinely.

Basic demographic characteristics, comorbid diseases (DM, hypertension, COPD, cerebrovascular accident [CVA], pectus deformity, any diagnosed malignancy, immunosuppressive medication taken for any reason), preoperative and first postoperative week laboratory parameters, European System for Cardiac Operative Risk Evaluation (EuroSCORE) II values, surgical procedure details (operation type, redo operation ratio, cross-clamping [XCL] time, cardiopulmonary bypass [CPB] time, Robicsek ratio, left internal thoracic artery [LITA] use, bleeding amount, intraoperative and postoperative red blood cells, and fresh frozen plasma), and postoperative complications (postoperative exploration, delayed chest closure, surgical subxiphoid decompression, superficial sternal wound infection [SSWI], DSWI, vacuum-assisted closure [VAC] usage, intubation time, intensive care unit [ICU] stay, hospital stay, and mortality) of all patients were recorded by reviewing medical records. The Centers for Disease Control and Prevention (or CDC) criteria were used for the definition of SSWI and DSWI^[6]^. Then, preoperative and perioperative data, postoperative complications, and sternal complications were compared between these three groups.

This study was approved by the Istanbul Basaksehir Cam and Sakura City Hospital Ethics Committee (Decision no: 2024-71).

### Statistics

Data were analyzed by using IBM SPSS Statistics for Windows, version 20.0 (IBM Corp., Armonk, N.Y., USA). Continuous variables in the study were presented as minimum, maximum, median, and interquartile range. Categorical variables were expressed as numbers and percentages. The normality of distribution was assessed by the Kolmogorov-Smirnov test. For numerical variables, the Kruskal-Wallis test was used to make comparisons between groups. Categorical variables were analyzed using the Pearson χ2 test and Fisher’s exact test. The Multivariate Analysis of Variance (MANOVA) was used to investigate the relationship between SSWI and DSWI and sternal closure techniques. The level of statistical significance was set at *P* < 0.05.

## RESULTS

The patients’ demographics, comorbid diseases, and laboratory parameters are shown in Table 1. The mean age was 58.5 ± 11.1 years, and 470 (72.9%) patients were male. The mean follow-up period was 245 ± 126 days (median: 241, 0 - 486 days). The distribution ratio of the groups is 141 (22%), 224 (35%), and 280 (43%) in Groups 1, 2, and 3, respectively. There was no significant difference between the groups in terms of basic demographic characteristics. Only in Group 2 were there fewer men (*P* = 0.03). There was also no significant difference between the groups in terms of comorbid diseases (DM, hypertension, COPD, CVA, pectus deformity, immunosuppressive medication). Only in Group 2, malignancy rates were higher than in the others (*P* = 0.03). Median EuroSCORE II was lower in Group 3 than in the other groups (2.3%, 2.8%, and 1.8% in Groups 1, 2, and 3, respectively, *P* = 0.03). There was no significant difference between the groups in terms of preoperative and postoperative laboratory parameters, except for hematocrit values. Preoperative and postoperative hematocrit values were lower in Group 1 than in the other groups (*P* = 0.02 and *P* = 0.006).

**Table 1:** Patients’ demographics, comorbid diseases, and laboratory parameters, and comparison between groups.

	Group 1 (n = 141)		Group 2 (n = 224)		Group 3 (n = 280)		
	Min-max or n (%)	Median (IQR)	Min - max or n (%)	Median (IQR)	Min - max or n (%)	Median (IQR)	*P*-value
Demographic data							
Sex, male	107 (75.9)		149 (66.5)		214 (76.4)		0.03^*^
Age (years)	19 - 79	59 (14)	18 - 81	60 (14)	23 - 86	59 (15)	0.75
Height (cm)	145 - 190	168 (12)	95 - 187	168 (14)	142 - 196	170 (10)	0.12
Weight (kg)	45 - 130	80 (16)	39 - 127	78 (20)	48 - 150	80 (19)	0.23
BSA (kg/m^2^)	1.40 - 2.37	1.88 (0.26)	1.22 - 2.42	1.86 (0.24)	1.4 - 2.6	1.9 (0.21)	0.06
BMI (m^2^)	15.9 - 55.6	27.6 (5.2)	15.6 - 110.8	27.7 (6.9)	20.2 - 46.2	27.5 (6.2)	0.86
Comorbid diseases							
Diabetes mellitus	55 (39.0)		90 (40.2)		107 (38.2)		0.90
Hypertension	64 (45.4)		126 (56.3)		145 (51.8)		0.13
COPD	12 (8.5)		22 (9.8)		24 (8.6)		0.86
CVA	10 (7.1)		23 (10.3)		25 (8.9)		0.58
Malignancy	5 (3.5)		16 (7.1)		1 (0.4)		0.03^*^
Pectus deformities	0		1 (0.4)		1 (0.4)		0.74
Immunosuppression	2 (1.4)		5 (2.2)		4 (1.4)		0.75
Ejection fraction (%)	22 - 60	55 (11)	15 - 65	55 (15)	20 - 65	55 (10)	0.25
EuroSCORE II	0.6 - 42.4	1.8 (2.3)	0.6 - 48.4	1.7 (2.8)	0.6 - 86.1	1.4 (1.8)	0.04^*^
Preoperative laboratory parameters							
WBC (109/L)	2.6 - 21.3	8.3 (3.3)	3.2 - 42.0	8.1 (3.1)	1.9 - 18.3	8 (1.9)	0.77
Hematocrit (%)	20.5 - 54.0	39.2 (8)	22.0 - 51.9	40.6 (7.3)	21.7 - 58.7	40.8 (6.4)	0.02^*^
Platelets (109/L)	74 - 528	230 (92.5)	34 - 522	236 (101)	63 - 688	235 (92)	0.80
Urea (mg/dL)	7.8 - 114.0	34.6 (17.1)	10.7 - 157	35 (17.1)	9.3 - 272	34.1 (15.3)	0.81
Creatinine (mg/dL)	0.3 - 10.9	0.93 (0.37)	0.47 - 5.6	0.93 (0.28)	0.07 - 9.02	0.93 (0.28)	0.98
Sodium (mEq/L)	122 - 145	139 (4)	123 - 149	139 (4)	124 - 146	139 (3)	0.16
Potassium (mEq/L)	3.2 - 5.8	4.4 (0.5)	2.2 - 5.9	4.3 (0.59)	2.6 - 5.7	4.3 (0.56)	0.87
ALT (IU/L)	3 - 301	17 (13)	5 - 222	19 (12.5)	1 - 338	18 (12)	0.45
AST (IU/L)	8 - 265	20 (12)	4 - 431	21 (11)	9 - 316	20 (9)	0.32
CRP (mg/dL)	0.2 - 165	3.9 (8.3)	0.3 - 162	3.6 (8.3)	0.2 - 259.0	3.7 (8.4)	0.90
Hba1c (mmol/mol)	4.2 - 14.3	6.1 (1.5)	2.9 - 13.9	5.9 (1.2)	4.6 - 13.4	5.9 (1.4)	0.69
Postoperative laboratory parameters							
WBC (109/L)	6.7 - 40	15.5 (7)	5.2 - 72.0	15.8 (9)	4.6 - 48.4	16.3 (8.1)	0.51
Hematocrit (%)	16.0 - 44.1	28 (5.2)	19 - 46.5	28 (5.4)	19.7 - 47.1	29.7 (6.2)	0.006^*^
Platelets (109/L)	56 - 480	167 (82)	21 - 445	167 (7.9)	38.6 - 496	175 (77)	0.31
Urea (mg/dL)	14 - 170	41.1 (20.5)	13.5 - 174	41.3 (17.3)	18 - 200	41.6 (19)	0.92
Creatinine (mg/dL)	0.4 - 7.8	1.1 (0.48)	0.54 - 6.2	1.2 (0.47)	0.6 - 3.1	1.25 (0.45)	0.88
Sodium (mEq/L)	133 - 163	142 (4)	134 - 163	143 (3)	126 - 154	143 (4)	0.07
Potassium (mEq/L)	2.8 - 5.7	4.2 (0.69)	2.7 - 5.7	4.2 (0.66)	2.6 - 5.6	4.2 (0.67)	0.87
ALT (IU/L)	3 - 528	23 (18)	2 - 850	23 (17.8)	4 - 711	22 (17)	0.57
AST (IU/L)	11 - 726	64 (53.5)	81.571	53.5 (38.5)	8 - 2512	59 (45)	0.25
CRP (mg/dL)	2.9 - 315.0	36.8 (25.9)	7 - 373	38.7 (26.9)	4.3 - 237.0	36.2 (27.4)	0.49

ALT=alanine aminotransferase; AST=aspartate aminotransferase; BMI=body mass index; BSA=body surface area; COPD=chronic obstructive pulmonary disease; CRP=C-reactive protein; CVA=cerebrovascular accident; EuroSCORE=European System for Cardiac Operative Risk Evaluation; IQR=interquartile range; WBC=white blood cells

Operative data is shown in Table 2. No difference was found between the groups in terms of the type of operation. Only the tricuspid ring annuloplasty rate was found to be higher in Group 2 than in the other groups (*P* = 0.04). In patients who underwent coronary artery bypass grafting (CABG), the use of LITA was significantly lower in Group 2 than in the other groups (85 [60.3%], 111 [49.6%], and 172 [61.4%] in Groups 1, 2, and 3, respectively, *P* = 0.01). In patients with left ventricular assist devices, no significant comparison could be made since only simple wire was used. No difference was found between the groups in terms of redo operation and Robicsek usage rates. No difference was found between the groups in terms of XCL time and CPB time.

**Table 2:** Operative data and comparison between groups.

	Group 1 (n = 141)		Group 2 (n = 224)		Group 3 (n = 280)		
	Min - max or n (%)	Median (IQR)	Min - max or n (%)	Median (IQR)	Min - max or n (%)	Median (IQR)	*P*-value
Infective endocarditis	5 (3.5)		11 (4.9)		9 (3.2)		0.98
Thoracic surgery	8 (5.7)		17 (7.6)		26 (9.3)		0.42
AVR	27 (19.1)		53 (23.7)		46 (16.4)		0.12
MVR	21 (14.9)		47 (21.0)		40 (14.3)		0.10
MRA	7 (5.0)		7 (3.1)		6 (2.1)		0.28
TVR	3 (2.1)		5 (2.2)		7 (2.5)		0.96
TRA	4 (2.8)		18 (8.0)		11 (3.9)		0.04^*^
PVR	1 (0.7)		1 (0.4)		1 (0.4)		0.88
Atrial septal defect	5 (3.5)		10 (4.5)		16 (5.7)		0.59
Morrow	0		1 (0.4)		2 (0.7)		0.59
CABG	101 (71.6)		139 (62.1)		193 (68.9)		0.11
Beating heart	6 (4.3)		10 (4.5)		10 (3.6)		0.87
The number of grafts	0 - 5	3 (3)	0 - 7	2 (3)	0 - 5	2 (3)	0.19
LITA graft	85 (60.3)		111 (49.6)		172 (61.4)		0.01^*^
LVAD	2 (1.4)		0		0		0.02^*^
Redo	8 (5.7)		19 (8.5)		16 (5.7)		0.40
XCL time (min)	0 - 323	90 (70)	0 - 343	93 (63)	0 - 314	86 (61)	0.21
CPB time (min)	0 - 444	137 (72)	0 - 428	136.5 (72)	0 - 515	132 (65)	0.22
Robicsek	1 (0.7)		2 (0.9)		2 (0.7)		0.97
Intraoperative RBC using	0 - 14	0 (2)	0 - 14	0 (2)	0 - 10	0 (2)	0.15
Intraoperative FFP using	0 - 4	0 (0)	0 - 4	0 (0)	0 - 4	0 (0)	0.99
Total amount of bleeding	50 - 4050	650 (500)	50-5200	650 (500)	50 - 5200	700 (450)	0.28
Postoperative RBC using	0 - 16	0 (2)	0 - 13	0 (1)	0 - 16	0 (1)	0.58
Postoperative FFP using	0 - 9	0 (1)	0 - 12	0 (1)	0 - 12	0 (1)	0.85

AVR=aortic valve replacement; CABG=coronary artery bypass grafting; CPB=cardiopulmonary bypass; FFP=fresh frozen plasma; IQR=interquartile range; LITA=left internal thoracic artery; LVAD=left ventricular assist device; MRA=mitral ring annuloplasty; MVR=mitral valve replacement; PVR=pulmonary valve replacement; RBC=red blood cells; TRA=tricuspid ring annuloplasty; TVR=tricuspid valve replacement; XCL=cross-clamping

Postoperative data are shown in Table 3. There was no difference between the groups in terms of delayed chest closure, subxiphoid decompression, SSWI, DSWI, VAC usage, intubation time, and mortality. Postoperative exploration was significantly lower in Group 3 than in the other groups (24 [17%], 26 [16%], and 17 [6.1%] in Groups 1, 2, and 3, respectively, *P* = 0.002). The hospital stay was found to be significantly lower in Group 3 compared to the other groups (*P* = 0.02). Median ICU days were higher in Group 1 than in other groups, although there was no statistical difference (3-2-2 days in Groups 1, 2, and 3, respectively, *P* = 0.051). Of all patients, 19 (2.9%) had DSWI, and 67 (10.4%) had SSWI. Table 4 also showed that there was no significant difference between the groups in terms of SSWI and DSWI according to Kruskal-Wallis test results (*P* = 0.84 and *P* = 0.98, respectively). MANOVA was used to investigate the relationship between SSWI, DSWI, and sternal closure techniques. We found that the three sternal closure techniques in our study did not have a statistically significant effect on SSWI and DSWI (*P* = 0.97).

**Table 3:** Postoperative data, and comparison between groups.

	Group 1 (n = 141)		Group 2 (n = 224)		Group 3 (n = 280)		
	Min-max or n (%)	Median (IQR)	Min-max or n (%)	Median (IQR)	Min-max or n (%)	Median (IQR)	*P*-value
Postoperative exploration	24 (17.0)		26 (11.6)		17 (6.1)		0.002^*^
Delayed chest closure	2 (1.4)		2 (0.9)		4 (1.4)		0.84
Subxiphoid decompression	1 (0.7)		3 (1.3)		5 (1.8)		0.67
SSWI	16 (11.3)		24 (10.7)		27 (9.6)		0.84
DSWI	4 (2.8)		7 (3.1)		8 (2.9)		0.98
VAC usage	8 (5.7)		12 (5.4)		11 (3.9)		0.65
Intubation time (hours)	2 - 1440	10 (9)	2 - 1440	11 (10)	3 - 672	10 (7)	0.14
ICU stay (days)	1 - 120	3 (2)	jan.-90	2 (2)	jan.-28	2 (1)	0.051
Hospital stay (days)	1 - 120	8 (7)	1 - 124	7 (4)	1 - 109	7 (3)	0.02^*^
Mortality	14 (9.9)		18 (8.0)		15 (5.4)		0.20

DSWI=deep sternal wound infection; ICU=intensive care unit; IQR=interquartile range; SSWI=superficial sternal wound infection; VAC=vacuum-assisted closure

**Table 4:** Comparison of groups with Kruskal-Wallis test in terms of sternal wound infections.

	N	Mean Rank	χ^^2^^	*P*-value
SSWI				
Group 1	141	326.1	0.33	0.84
Group 2	224	324.5		
Group 3	280	320.6		
DSWI				
Group 1	141	322.6	0.03	0.98
Group 2	224	323.5		
Group 3	280	322.7		

DSWI=deep sternal wound infection; SSWI=superficial sternal wound infection

## DISCUSSION

In today's world where small incision operations are becoming more common, one of the most important disadvantages of median sternotomy compared to thoracotomy is sternal wound infections and complications. Sternal complications can be SSWI, which involves the skin and subcutaneous tissue, or DSWI, which can be life-threatening, such as mediastinitis^[7]^. It is an important cause of morbidity and mortality, as well as prolonged ICU and hospital stay after cardiac surgery^[5]^. It is also an important problem in terms of cost due to repeated reoperations, debridement, VAC use, and reconstruction procedures. Many risk factors such as obesity, COPD, DM, immunosuppression, osteoporosis, advanced age, redo surgery, bleeding, and prolonged ventilation have been reported^[4^,^5^,^7^,^8]^. Although patient-related risk factors are important, they are often unchangeable risk factors. It is also important to identify risk factors that may be related to surgical technique. Therefore, the relationship between sternum closure techniques, which are modifiable risk factors, and this complication should be clarified. Many closure techniques have been used for sternotomy, which has been used since the 1950s. Many materials such as sternum wires, cables, plates, steel bands, polydioxanone sutures, nylon bands, and custom-made plates have also been used^[8]^. Steel wires are most commonly used because they are fast, easily accessible, and low-cost. Keating et al. even reported the use of wires as a superior sternal repair technique, considering the lower cost profile of wires compared to sternal plating with similar sternal outcomes^[9]^. There are many publications on which configuration these wires should be in the sternum and which technique is superior^[4^,^5^,^8^,^10^,^11]^. The most commonly used techniques are simple wire and figure-of-8, but their combinations are also used. The vast majority of studies conducted so far have compared these two most commonly used techniques. In this study, we compared and evaluated the third technique, which is a combination of these techniques that is routinely used in our clinic and has not been sufficiently evaluated in previous studies.

One of the important points of this study is that, unlike many previous studies, not only the patient group with sternal complications but also all patients who underwent surgery during the study period were evaluated in detail in terms of all risks. As seen in Table 1, the basic demographic characteristics of the patient groups such as age, height, weight, body mass index, and comorbid conditions such as hypertension, DM, COPD, renal function, immunosuppression, and basic preoperative laboratory parameters (except hematocrit) are similar. Only the male sex ratio was lower in Group 2 than in the other groups, and the number of patients diagnosed with any malignancy was higher in Group 2 than in the others. Also, EuroSCORE II values were lower in Group 3 than in the other groups. Similarly, as seen in Table 2, the types of operations between the groups, XCL and CPB times, bleeding amounts, redo operations, and intraoperative and postoperative blood products used are similar. Only LITA use was lower in Group 2 compared to the others. As seen in Table 3, no difference was found between the groups in terms of delayed chest closure and subxiphoid decompression. However, postoperative exploration rates were higher in Group 1 than in the other groups. We can say that the basic risk factors of the patients are similar between the groups, although not the same. This analysis, performed with this relatively high number of patients, can provide clearer information than other studies in terms of showing the relationship between sternal closure technique and sternal complications.

In our study, the incidence of SSWI in all patients was found to be 67 (10.3%). However, the incidence of DSWI in all patients was found to be 19 (2.9%). This rate is similar to other studies in the literature and is seen between 0.5% and 6.8%^[7]^. In the comparison between the groups, we did not find any statistical difference in terms of DSWI (2.8%, 3.1%, 2.9% in Groups 1, 2, and 3, respectively). Similarly, we did not find any statistical difference between the groups for SSWI (11.3%, 10.7%, and 9.6% in Groups 1, 2, and 3, respectively). We also did not find any difference in terms of VAC use. With these results, we can say that there is no difference in terms of sternal complications with these three sternal closure techniques. In the study of Tekümit et al.^[5]^, unlike our study, only two groups were compared, but similarly, no difference was found between the groups. In contrast, Almdahl et al.^[4]^ found that figure-of-8 was more beneficial than simple wire, again comparing only two groups. Asghar et al.^[12]^ also compared the two groups and reported that they did not find any significant difference between figure-of-8 and simple wire. Khasati et al.^[13]^ also reported that figure-of-8 was not superior to simple wire. The results in our study are similar to these three studies in terms of these two groups. However, it provides important results in terms of showing that these techniques can also be used in combination, and that the results for sternal complications are similar to these other frequently used techniques. In addition, in the literature, the double wire technique is also performed in addition to simple wire. Better results have been reported with double wire than simple wire^[10^,^11]^. Since double wire was not used in our patients, a comparison could not be made, but it would be useful to investigate this in future studies.

Bilateral internal thoracic artery (BITA) use is associated with increased DSWI^[14]^. In our study, it was observed that LITA use was less frequent in Group 2 than in the others. Since BITA use is not common in our clinic, a comparison could not be made in terms of BITA. However, in the groups where LITA use was more frequent, sternal complications were found to be similar to the other group. Since there was no relationship between LITA use alone and an increase in sternal complications, it is of utmost importance to use at least one arterial graft for CABG.

In normal respiratory dynamics, bone stability is effectively provided with the simple wire technique. However, in cases such as asymmetrical respiratory patterns, inappropriate movements of the patient and lifting one arm, and leaning on one chest, sternum stability can be better provided with figure-of-8 because both lateral and longitudinal movements can be better restricted with this technique^[15]^. However, one of the most important disadvantages of this technique is that even if a single wire breaks, it can cause sternal instability because the connection of two wires will be separated. To reduce this risk, the manubrium sterni, which is the most important part in terms of sternum stability, can be wired in the form of a figure of 8, and the remaining wires can be wired in the form of simple wires. The comparison of this technique with others is the main result of this study and the main point that distinguishes it from other studies. It has been observed that this technique also has similar results for sternal complications to other techniques. Any of these techniques can be used according to the surgeon's knowledge, experience, and preference.

Another point is the number of sternal wires in the figure-of-8 technique. It has been reported that sternal stability can be better achieved with five figure-of-8 wires instead of four^[4]^. Although the DSWI rates in our study were generally similar to other studies, they were found to be significantly lower in this study, at 0.06%^[4]^. This shows the importance of closing the lower part of the sternum, which is a sensitive area in terms of separation, and that the use of five wires may be better. However, this finding needs to be supported by further studies.

Sternal complications cause prolonged hospital stay^[16]^. ICU and hospital stays were found to be longer than those in patients without DSWI due to reasons such as repeated interventions and VAC use. ICU stay was longer in Group 1, although there was no statistical difference. We believe that the main reason for this is that there was significantly more postoperative exploration in Group 1 than in the other groups. However, hospital stays were found to be shorter in Group 3 than in the other groups. The main reason for this may be that the patients in Group 3 had lower EuroSCORE II values and less postoperative exploration compared to the other groups. Therefore, future studies in a patient group matched for these risks would be useful. However, with the current results in our study, although there is no difference between the groups in terms of sternal complications, the combined technique may be more beneficial than the others in terms of hospital stay.

In-hospital mortality after cardiac surgery has been reported to be approximately 3.4%^[17]^. Considering that DSWI increases morbidity and mortality, mortality in our study was higher than standard cardiac surgery, but we did not find a statistical difference between the groups (9.9%, 8.8%, and 5.4%, respectively).

### Limitations

The first limitation of this study is that it is a retrospective single-center study. Secondly, all patients who underwent cardiac surgery, not isolated CABG, were included. Therefore, it was a heterogeneous patient group, especially in terms of LITA use. Third, although many risk factors for sternal complications were compared between the groups and were generally similar, the groups did not have the same characteristics. In particular, the lower EuroSCORE II and postoperative exploration rates in Group 3 may have affected these results. Future prospective studies with patients with similar characteristics will provide more accurate findings on this issue. Fourth, in terms of DSWI, reasons such as infection and culture results could not be evaluated. Finally, considering that osteoporosis is an important risk factor, it could not be evaluated because there is not enough data about its frequency and degree. However, cables were routinely used in our clinic for patients with poor bone structure or obesity, and all patients who used cables were excluded from the study.

## CONCLUSION

In patients undergoing cardiac surgery, we found no difference in sternal complications (DSWI, SSWI) between the three most commonly used closure techniques (simple wire, figure-of-8, and their combination). We found that the length of hospital stay was shorter in patients with the combined technique than in those with the other two techniques. However, this needs to be investigated in future prospective studies.

## Data Availability

The authors declare that the hospital’s database was used for the data and is not publicly available. However, it can be provided to institutions upon request.

## Artificial Intelligence Usage

The authors declare that no artificial intelligence tool was used in the preparation of this article.
